# Crystal structure and Hirshfeld surface analysis of 2-(4-bromo­phen­yl)-4-methyl-6-oxo-1-phenyl-1,6-di­hydro­pyridine-3-carbo­nitrile

**DOI:** 10.1107/S2056989022006466

**Published:** 2022-07-05

**Authors:** Farid N. Naghiyev, Victor N. Khrustalev, Ekaterina V. Dobrokhotova, Mehmet Akkurt, Ali N. Khalilov, Ajaya Bhattarai, İbrahim G. Mamedov

**Affiliations:** a Department of Chemistry, Baku State University, Z. Khalilov str. 23, Az, 1148, Baku, Azerbaijan; b Peoples’ Friendship University of Russia (RUDN University), Miklukho-Maklay St., 6, Moscow, 117198, Russian Federation; c N. D. Zelinsky Institute of Organic Chemistry RAS, Leninsky Prosp. 47, Moscow, 119991, Russian Federation; d Department of Physics, Faculty of Sciences, Erciyes University, 38039 Kayseri, Turkey; e "Composite Materials" Scientific Research Center, Azerbaijan State Economic University (UNEC), H. Aliyev str. 135, Az 1063, Baku, Azerbaijan; f Department of Chemistry, M.M.A.M.C (Tribhuvan University) Biratnagar, Nepal; Katholieke Universiteit Leuven, Belgium

**Keywords:** crystal structure, 1,6-di­hydro­pyridine, hydrogen bond, C—Br⋯π inter­actions, Hirshfeld surface analysis

## Abstract

In the title compound, C_19_H_13_BrN_2_O, the pyridine ring is essentially planar [maximum deviation = 0.024 (4) Å for the N atom] and makes dihedral angles of 74.6 (2) and 65.8 (2)°, respectively, with the phenyl and bromo­phenyl rings, which subtend a dihedral angle of 63.1 (2)°. In the crystal, mol­ecules are connected along the *c*-axis direction *via* C—Br⋯π inter­actions, generating zigzag chains parallel to the (010) plane. C—H⋯N and C—H⋯O hydrogen-bonding inter­actions further connect the mol­ecules, forming a three-dimensional network and reinforcing the mol­ecular packing. Hirshfeld surface analysis indicates that the most important contributions to the crystal packing are from H⋯H (36.2%), C⋯H/H⋯C (21.6%), N⋯H/H⋯N (12.2%), and Br⋯H/H⋯Br (10.8%) inter­actions.

## Abstract 2

Mol­ecules in the crystal are connected along the *c*-axis direction by C—Br⋯π inter­actions, resulting in zigzag chains parallel to the (010) plane. C—H⋯N and C—H⋯O hydrogen-bonding inter­actions further connect the mol­ecules, forming a three-dimensional network and reinforcing the mol­ecular packing.

## 1. Chemical context

**Figure 1:**
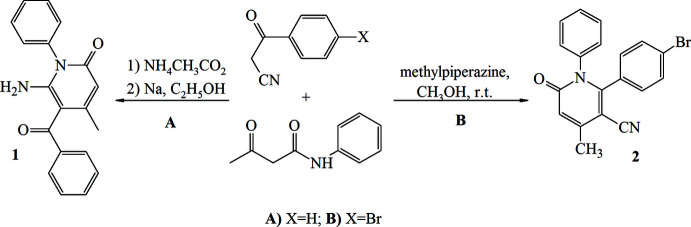
The reaction of acetoacetanilide with 3-oxo-3-aryl­propane­nitriles.

C—C and C—N bond-forming reactions are a cornerstone of organic synthesis, materials science and medicinal chemistry (Zubkov *et al.*, 2018 ▸; Shikhaliyev *et al.*, 2019 ▸; Viswanathan *et al.*, 2019 ▸; Gurbanov *et al.*, 2020 ▸). Nitro­gen heterocycles, particularly those including the 2-pyridone core, play a key role in medicinal chemistry and natural product synthesis (Sośnicki & Idzik, 2019 ▸; Duruskari *et al.*, 2020 ▸; Sangwan *et al.*, 2022 ▸). We report herein the synthesis of 2-pyridone, **2**, on the basis of a one-step reaction of acetoacetanilide with 3-(4-bromo­phen­yl)-3-oxo­propane­nitrile (Path **B**). Under two-step reaction conditions (Fig. 1 ▸), the inter­action of acetoacetanilide with 3-oxo-3-phenyl­propane­nitrile led to the formation of another 2-pyridone, **1** (Path **A**), reported in the literature (Wardakhan & Agami, 2001 ▸). 
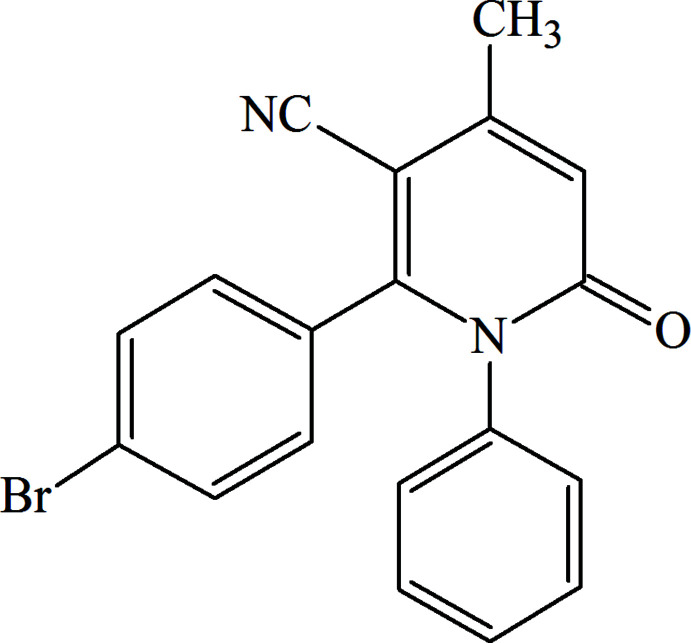


Thus, in the framework of our ongoing structural studies (Naghiyev *et al.*, 2020 ▸, 2021 ▸, 2022 ▸; Khalilov *et al.*, 2022 ▸), we report the crystal structure and Hirshfeld surface analysis of the title compound, 2-(4-bromo­phen­yl)-4-methyl-6-oxo-1-phenyl-1,6-di­hydro­pyridine-3-carbo­nitrile.

## 2. Structural commentary

**Figure 2:**
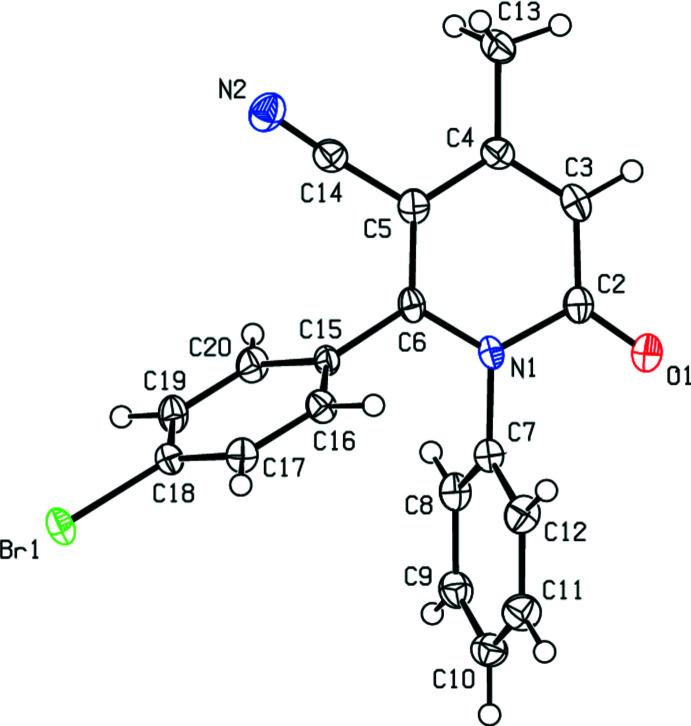
The mol­ecular structure of the title compound. Displacement ellipsoids are drawn at the 50% probability level.

In the title compound, (Fig. 2 ▸), the pyridine ring (N1/C2–C6) is largely planar [maximum deviation = 0.024 (4) Å for N1]. The phenyl and bromo­phenyl groups are linked to the central pyridine ring in an equatorial arrangement. The pyridine ring subtends dihedral angles of 74.6 (2) and 65.8 (2)° with the phenyl (C7–C12) and bromo­phenyl (C15–C20) rings, which in turn make a dihedral angle of 63.1 (2)° with each other.

## 3. Supra­molecular features and Hirshfeld surface analysis

**Figure 3:**
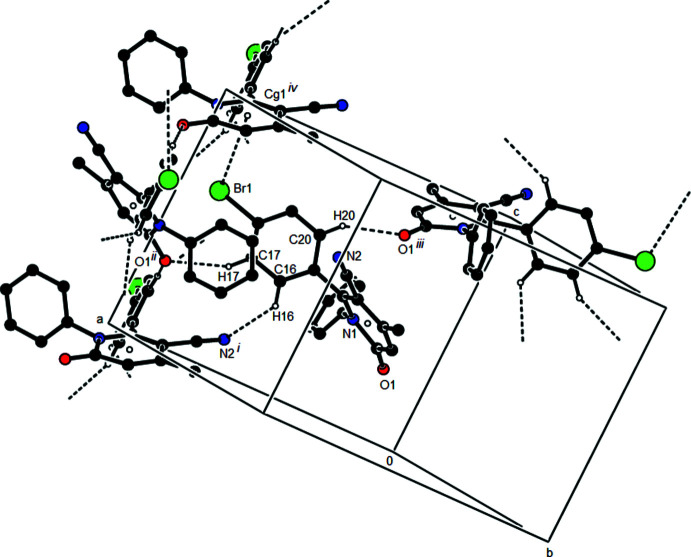
A general view of the C—H⋯N, C—H⋯O hydrogen bonds and C—Br⋯π inter­actions of the title compound. Symmetry codes: (i) *x* + 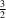 , −*y* − 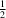 , *z* − 1; (ii) −*x* + 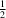 , *y* + 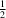 , *z* + 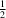 ; (iii) −*x* + 1, −*y* + 1, *z* + 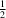 ; (iv) 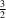 − *x*, − 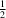 + *y*, 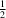 + *z*.

**Figure 4:**
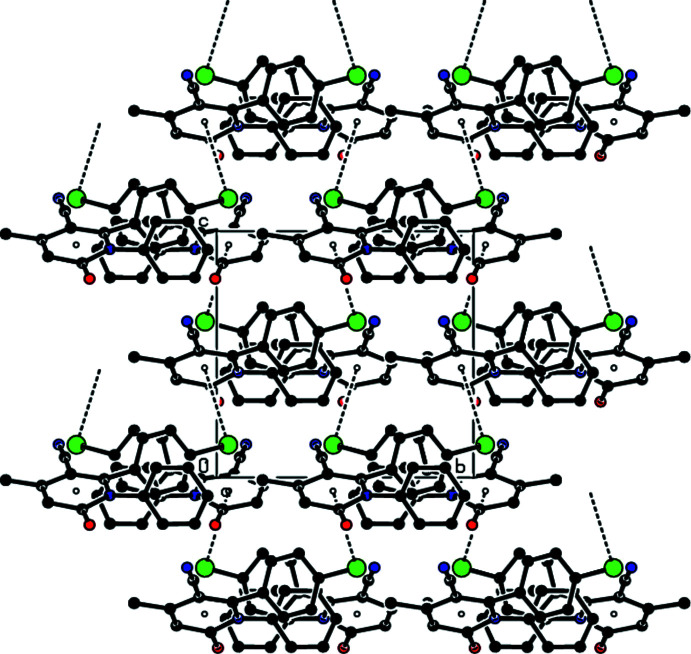
Packing view of the title compound along the *a* axis showing the C—Br⋯π inter­actions as dashed lines.

Fig. 3 ▸ shows a general view of the C—H⋯N and C—H⋯O hydrogen bonds (Table 1 ▸) and C—Br⋯π inter­actions in the unit cell of the title compound. In the crystal, mol­ecules are joined along the *c*-axis direction by C—Br⋯π inter­actions [C18—Br1⋯*Cg*1^iv^: C18—Br1 = 1.944 (4) Å, Br1⋯*Cg*1^iv^ = 3.4788 (18) Å, C18⋯*Cg*1^iv^ = 4.283 (5) Å, C18—Br1⋯*Cg*1^iv^ = 100.50 (13)°; *Cg*1 is the centroid of the N1/C2–C6 pyridine ring; symmetry code: (iv) *x* + 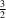 , −*y* − 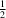 , *z*], generating zigzag chains parallel to the (010) plane (Figs. 4 ▸ and 5 ▸). C—H⋯N and C—H⋯O hydrogen bonds link these mol­ecules, establishing a three-dimensional network and strengthening the mol­ecular packing.

**Figure 6:**
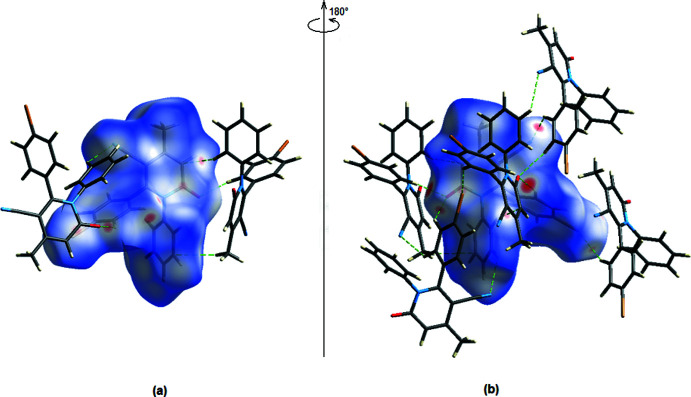
(*a*) Front and (*b*) back sides of the three-dimensional Hirshfeld surface of the title compound mapped over *d*_norm_, with a fixed colour scale of −0.2437 to 1.2589 a.u.

*CrystalExplorer17.5* (Turner *et al.*, 2017 ▸) was used to analyse and visualize the inter­molecular inter­actions of the title compound. Fig. 6 ▸ *a*,*b* depicts the front and back sides of the Hirshfeld surface plotted over *d*_norm_ in the range of −0.2437 to 1.2589 a.u. The red spots on the Hirshfeld surface indicate C—H⋯N and C—H⋯O inter­actions (Table 1 ▸).

**Figure 7:**
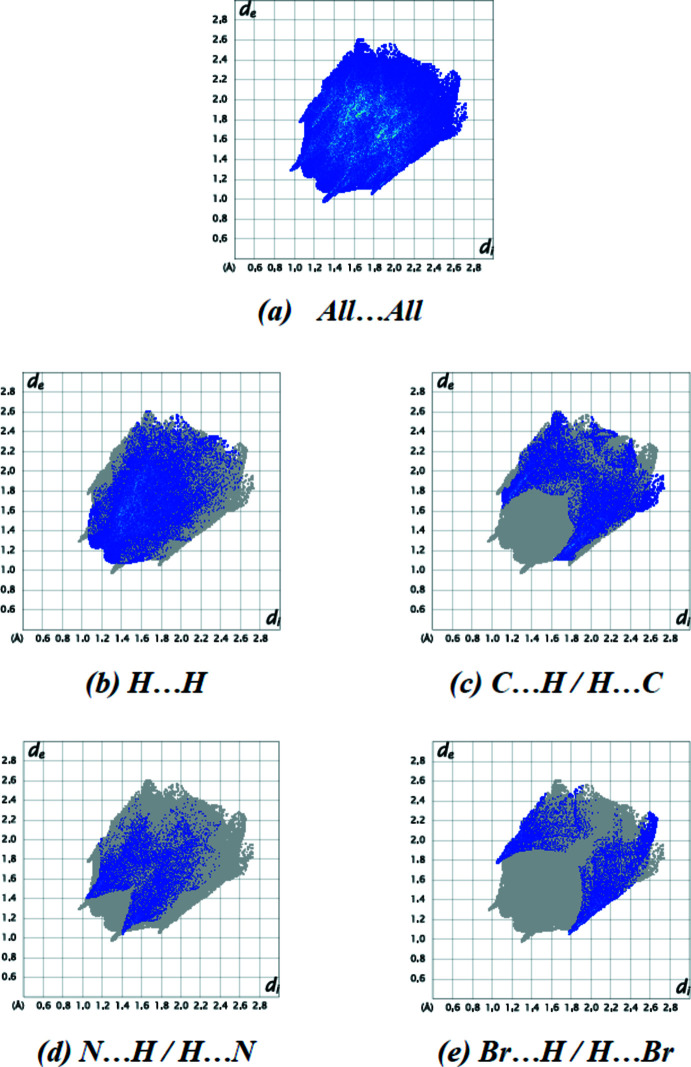
The two-dimensional fingerprint plots of the title compound, showing (*a*) all inter­actions, and delineated into (*b*) H⋯H, (*c*) C⋯H/H⋯C, (*d*) N⋯H/H⋯N and (*e*) Br⋯H/H⋯Br inter­actions. [*d*_e_ and *d*_i_ represent the distances from a point on the Hirshfeld surface to the nearest atoms outside (external) and inside (inter­nal) the surface, respectively].

The overall two-dimensional fingerprint plot for the title compound and those delineated into H⋯H (36.2%, Fig. 7 ▸ *b*), C⋯H/H⋯C (21.6%, Fig. 7 ▸ *c*), N⋯H/H⋯N (12.2%, Fig. 7 ▸ *d*), and Br⋯H/H⋯Br (10.8%, Fig. 7 ▸ *e*) inter­actions, as well as their relative contributions to the Hirshfeld surface, are shown in Fig. 7 ▸, while Tables 1 ▸ and 2 ▸ provide data on the distinct inter­molecular contacts. The remaining weak inter­actions (contribution percentages) are O⋯H/H⋯O (7.2%), Br⋯C/C⋯Br (3.6%), C⋯C (3.0%), Br.·N/N⋯Br (2.2%), O⋯C/C⋯O (2.2%) and Br⋯O/O⋯Br (0.8%), these contacts having little directional influence on the packing.

## 4. Database survey

A search of the Cambridge Structural Database (CSD version 5.42, updated September 2021; Groom *et al.*, 2016 ▸) for the basic skeleton of 6-oxo-1,6-di­hydro­pyridine gave five compounds very similar to the title compound.

The cations in the crystal of FONDOC01 (Pérez-Aguirre *et al.*, 2015 ▸) inter­act with the anions through O—H⋯O and N—H⋯O hydrogen bonds, forming a three-dimensional supra­molecular network.

In the crystal of SECPUN (Thanigaimani *et al.*, 2012 ▸), an N—H⋯O hydrogen bond connects the cation and anion, while a pair of N—H⋯O hydrogen bonds connects the two anions with an 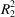 (8) ring motif. Weak N—H⋯O and C—H⋯O hydrogen bonds connect the aggregates, forming a three-dimensional network.

The ion pairs in the crystal of SUYXIU (Hemamalini & Fun, 2010 ▸) are linked by O—H⋯O, N—H⋯O, N—H⋯Br and C—H⋯O hydrogen bonds, producing a two-dimensional network parallel to the *bc* plane.

In the crystal of XOZCUL (Shishkina *et al.*, 2009 ▸), the pyridine-3-carboxyl­ate mol­ecules form layers parallel to (010), which are linked by hydrogen bonds mediated by the bridging solvate mol­ecules.

The asymmetric unit of GIHCOQ (Gupta *et al.*, 2007 ▸) contains four mol­ecules. The compound forms hydrogen-bonded sheets parallel to the [001] direction *via* inter­molecular N—H⋯O and O—H⋯O hydrogen bonds. Each sheet is made up of linked dimers generated by 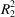 (8) N—H⋯O hydrogen-bonded motifs. Inter­molecular N—H⋯O and O—H⋯O hydrogen bonds generate sheets parallel to the [001] direction. Each sheet is made up of linked dimers formed by N—H⋯O hydrogen bonds with 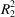 (8) motifs.

## 5. Synthesis and crystallization

To a solution of 3-(4-bromo­phen­yl)-3-oxo­propane­nitrile (1.14 g; 5.1 mmol) and acetoacetanilide (0.92 g; 5.2 mmol) in methanol (25 mL), methyl­pyperazine (3 drops) was added and the mixture was stirred at room temperature for 48 h. Then 15 mL of methanol were removed from the reaction mixture, which was left overnight. The precipitated crystals were separated by filtration and recrystallized from ethanol/water (1:1) solution (yield 49%; m.p. 484–485 K).

^1^H NMR (300 MHz, DMSO-*d*_6_, ppm): 2.21 (*s*, 3H, CH_3_); 6.61 (*s*, 1H, =CH); 7.19–7.89 (*m*, 9H, 9Ar—H). ^13^C NMR (75 MHz, DMSO-*d*_6_, ppm): 20.58 (CH_3_), 94.75 (=C_quat._), 116.17 (CN), 118.27 (=CH), 120.87 (2CH_arom._), 122.95 (Br—C_arom._), 125.30 (CH_arom._), 127.43 (C_arom._), 129.55 (2CH_arom._), 129.70 (2CH_arom._), 134.09 (2CH_arom._), 138.94 (C_arom._), 143.81 (=C_quat._), 153.68 (=C_quat_—N), 165.44 (C=O).

## 6. Refinement

Crystal data, data collection and structure refinement details are summarized in Table 3 ▸. All C-bound H atoms were placed at calculated positions and refined using a riding model, with C—H = 0.95 Å for aromatic H atoms and 0.98 Å for methyl H atoms, and with *U*_iso_(H) = 1.2 or 1.5*U*_eq_(C). Owing to poor agreement between observed and calculated intensities, nineteen outliers (8 4 0, 17 6 2, 13 9 2, 18 5 0, 9 11 2, 18 2 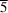 , 17 3 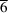 , 0 12 4, 4 11 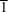 , 3 5 0, 18 5 2, 2 0 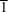 , 5 3 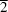 , 18 2 5, 15 8 2, 0 10 8, 5 3 2, 0 12 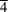 and 17 6 1) were omitted in the final cycles of refinement.

## Supplementary Material

Crystal structure: contains datablock(s) I. DOI: 10.1107/S2056989022006466/vm2267sup1.cif

Structure factors: contains datablock(s) I. DOI: 10.1107/S2056989022006466/vm2267Isup2.hkl

Supporting information file. DOI: 10.1107/S2056989022006466/vm2267Isup3.cml

CCDC reference: 2181245

Additional supporting information:  crystallographic information; 3D view; checkCIF report

## Figures and Tables

**Figure 5:**
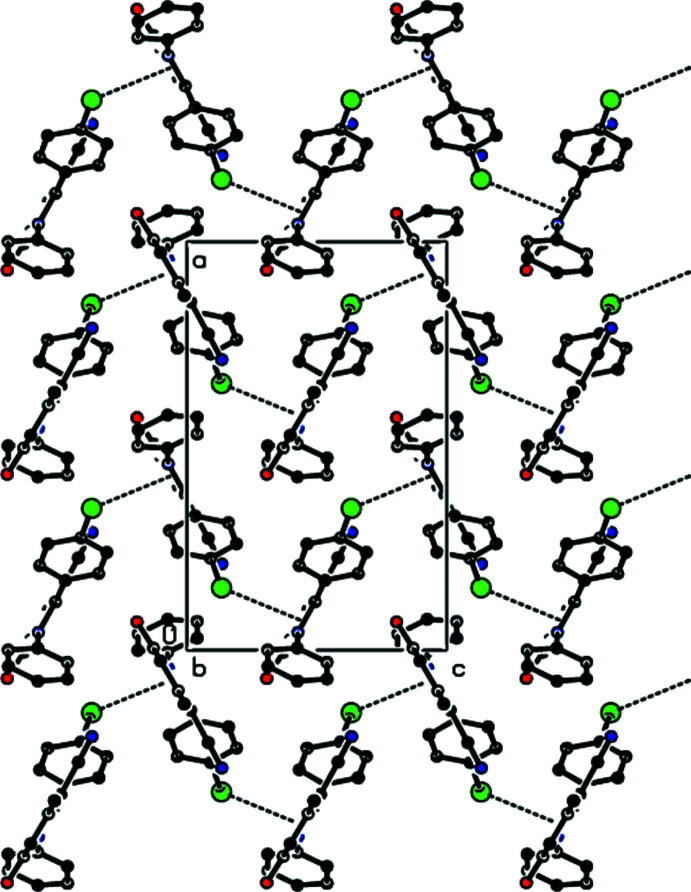
Packing view of the title compound along the *b* axis with the C—Br⋯π inter­actions indicated by dashed lines.

**Table 1:** Hydrogen-bond geometry (Å, °)

*D*—H⋯*A*	*D*—H	H⋯*A*	*D*⋯*A*	*D*—H⋯*A*
C16—H16⋯N2^i^	0.95	2.55	3.234 (6)	129
C17—H17⋯O1^ii^	0.95	2.56	3.342 (6)	140
C20—H20⋯O1^iii^	0.95	2.40	3.256 (6)	150

Symmetry codes: (i) 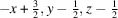 ; (ii) 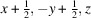 ; (iii) 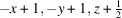 .

**Table 2:** Summary of short inter­atomic contacts (Å) in the title compound

Contact	Distance	Symmetry operation
H19⋯H13*B*	2.59	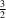 − *x*, − 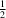 + *y*, 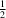 + *z*
H17⋯O1	2.56	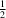 + *x*, 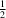 − *y*, *z*
O1⋯H20	2.40	1 − *x*, 1 − *y*, − 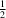 + *z*
N2⋯H16	2.55	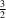 − *x*, 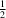 + *y*, 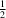 + *z*
C9⋯H11	2.99	1 − *x*, −*y*, 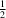 + *z*
C10⋯H13*A*	3.03	*x*, −1 + *y*, *z*

**Table 3:** Experimental details

Crystal data	
Chemical formula	C_19_H_13_BrN_2_O
*M*_r_	365.21
Crystal system, space group	Orthorhombic, *P* *n* *a*2_1_
Temperature (K)	100
*a*, *b*, *c* (Å)	15.58979 (16), 10.33883 (10), 9.91195 (9)
*V* (Å^3^)	1597.61 (3)
*Z*	4
Radiation type	Cu *K*α
μ (mm^−1^)	3.55
Crystal size (mm)	0.25 × 0.24 × 0.21

Data collection	
Diffractometer	XtaLAB Synergy, Dualflex, HyPix
Absorption correction	Multi-scan (*CrysAlis PRO*; Rigaku OD, 2021 ▸)
*T*_min_, *T*_max_	0.413, 0.462
No. of measured, independent and observed [*I* > 2σ(*I*)] reflections	45325, 3359, 3339
*R*_int_	0.047
(sin θ/λ)_max_ (Å^−1^)	0.648

Refinement	
*R*[*F* ^2^ > 2σ(*F* ^2^)], *wR*(*F* ^2^), *S*	0.037, 0.099, 1.05
No. of reflections	3359
No. of parameters	209
No. of restraints	1
H-atom treatment	H-atom parameters constrained
Δρ_max_, Δρ_min_ (e Å^−3^)	1.27, −0.89
Absolute structure	Flack *x* determined using 1531 quotients [(*I* ^+^)−(*I* ^−^)]/[(*I* ^+^)+(*I* ^−^)] (Parsons *et al.*, 2013 ▸).
Absolute structure parameter	−0.012 (18)

Computer programs: *CrysAlis PRO* (Rigaku OD, 2021 ▸), *SHELXT* (Sheldrick, 2015*a* ▸), *SHELXL* (Sheldrick, 2015*b* ▸), *ORTEP-3 for Windows* (Farrugia, 2012 ▸) and *PLATON* (Spek, 2020 ▸).

## supplementary crystallographic information

### Crystal data

**Table:**

C_19_H_13_BrN_2_O	*D*_x_ = 1.518 Mg m^−^^3^
*M**_r_* = 365.21	Cu *K*α radiation, λ = 1.54178 Å
Orthorhombic, *P**n**a*2_1_	Cell parameters from 35934 reflections
*a* = 15.58979 (16) Å	θ = 2.7–79.0°
*b* = 10.33883 (10) Å	µ = 3.55 mm^−^^1^
*c* = 9.91195 (9) Å	*T* = 100 K
*V* = 1597.61 (3) Å^3^	Prism, colourless
*Z* = 4	0.25 × 0.24 × 0.21 mm
*F*(000) = 736	

### Data collection

**Table:**

XtaLAB Synergy, Dualflex, HyPix diffractometer	3339 reflections with *I* > 2σ(*I*)
Radiation source: micro-focus sealed X-ray tube	*R*_int_ = 0.047
φ and ω scans	θ_max_ = 88.3°, θ_min_ = 5.1°
Absorption correction: multi-scan (CrysAlisPro; Rigaku OD, 2021)	*h* = −19→19
*T*_min_ = 0.413, *T*_max_ = 0.462	*k* = −12→12
45325 measured reflections	*l* = −12→12
3359 independent reflections	

### Refinement

**Table:**

Refinement on *F*^2^	Secondary atom site location: difference Fourier map
Least-squares matrix: full	Hydrogen site location: inferred from neighbouring sites
*R*[*F*^2^ > 2σ(*F*^2^)] = 0.037	H-atom parameters constrained
*wR*(*F*^2^) = 0.099	*w* = 1/[σ^2^(*F*_o_^2^) + (0.0658*P*)^2^ + 1.8045*P*] where *P* = (*F*_o_^2^ + 2*F*_c_^2^)/3
*S* = 1.05	(Δ/σ)_max_ < 0.001
3359 reflections	Δρ_max_ = 1.27 e Å^−^^3^
209 parameters	Δρ_min_ = −0.89 e Å^−^^3^
1 restraint	Absolute structure: Flack *x* determined using 1531 quotients [(*I*^+^)-(*I*^-^)]/[(*I*^+^)+(*I*^-^)] (Parsons *et al.*, 2013).
Primary atom site location: difference Fourier map	Absolute structure parameter: −0.012 (18)

### Special details

**Table:**

Experimental. CrysAlisPro 1.171.41.117a (Rigaku OD, 2021) Empirical absorption correction using spherical harmonics, implemented in SCALE3 ABSPACK scaling algorithm.
Geometry. All esds (except the esd in the dihedral angle between two l.s. planes) are estimated using the full covariance matrix. The cell esds are taken into account individually in the estimation of esds in distances, angles and torsion angles; correlations between esds in cell parameters are only used when they are defined by crystal symmetry. An approximate (isotropic) treatment of cell esds is used for estimating esds involving l.s. planes.

### Fractional atomic coordinates and isotropic or equivalent isotropic displacement parameters (Å^2^)

**Table:**

	*x*	*y*	*z*	*U*_iso_*/*U*_eq_	
Br1	0.84710 (3)	−0.04550 (4)	0.63282 (7)	0.02494 (16)	
O1	0.4307 (2)	0.4948 (4)	0.3074 (4)	0.0278 (7)	
N1	0.5437 (2)	0.4161 (4)	0.4275 (4)	0.0188 (7)	
N2	0.7885 (2)	0.6147 (4)	0.6339 (5)	0.0294 (7)	
C2	0.4948 (3)	0.5231 (5)	0.3709 (4)	0.0216 (10)	
C3	0.5263 (3)	0.6562 (5)	0.3980 (4)	0.0238 (9)	
H3	0.4934	0.7271	0.3657	0.029*	
C4	0.5993 (3)	0.6812 (4)	0.4665 (4)	0.0215 (8)	
C5	0.6468 (3)	0.5678 (5)	0.5140 (5)	0.0222 (10)	
C6	0.6185 (3)	0.4373 (4)	0.4937 (4)	0.0188 (8)	
C7	0.5051 (3)	0.2830 (4)	0.4247 (4)	0.0205 (8)	
C8	0.4705 (3)	0.2349 (5)	0.5395 (5)	0.0237 (9)	
H8	0.4733	0.2837	0.6205	0.028*	
C9	0.4295 (3)	0.1103 (5)	0.5393 (5)	0.0263 (9)	
H9	0.4052	0.0789	0.6209	0.032*	
C10	0.4243 (3)	0.0354 (5)	0.4255 (5)	0.0268 (10)	
H10	0.3970	−0.0467	0.4261	0.032*	
C11	0.4602 (3)	0.0848 (5)	0.3123 (5)	0.0290 (10)	
H11	0.4587	0.0354	0.2315	0.035*	
C12	0.5005 (3)	0.2097 (5)	0.3110 (5)	0.0260 (9)	
H12	0.5243	0.2417	0.2293	0.031*	
C13	0.6315 (3)	0.8212 (5)	0.4893 (5)	0.0274 (10)	
H13A	0.5887	0.8824	0.4551	0.041*	
H13B	0.6857	0.8338	0.4412	0.041*	
H13C	0.6403	0.8359	0.5859	0.041*	
C14	0.7262 (3)	0.5907 (4)	0.5820 (5)	0.0233 (9)	
C15	0.6711 (3)	0.3187 (4)	0.5311 (4)	0.0174 (8)	
C16	0.7071 (3)	0.2397 (4)	0.4329 (4)	0.0201 (8)	
H16	0.6956	0.2589	0.3409	0.024*	
C17	0.7600 (3)	0.1318 (4)	0.4629 (4)	0.0214 (8)	
H17	0.7843	0.0813	0.3924	0.026*	
C18	0.7751 (3)	0.1027 (4)	0.5913 (4)	0.0205 (8)	
C19	0.7397 (3)	0.1793 (5)	0.6909 (4)	0.0232 (9)	
H19	0.7505	0.1587	0.7828	0.028*	
C20	0.6878 (3)	0.2878 (4)	0.6599 (4)	0.0230 (9)	
H20	0.6647	0.3389	0.7307	0.028*	

### Atomic displacement parameters (Å^2^)

**Table:**

	*U*^11^	*U*^22^	*U*^33^	*U*^12^	*U*^13^	*U*^23^
Br1	0.0283 (2)	0.0232 (3)	0.0233 (2)	0.00633 (14)	−0.0028 (2)	0.0055 (2)
O1	0.0269 (16)	0.0306 (17)	0.0260 (16)	0.0013 (15)	−0.0068 (13)	0.0022 (14)
N1	0.0179 (16)	0.0204 (18)	0.0182 (17)	0.0037 (15)	−0.0015 (13)	0.0042 (14)
N2	0.0286 (17)	0.0326 (19)	0.0270 (17)	0.0030 (14)	−0.003 (2)	−0.009 (2)
C2	0.0214 (19)	0.027 (3)	0.016 (2)	0.0027 (19)	0.0010 (16)	0.0020 (17)
C3	0.024 (2)	0.024 (2)	0.024 (2)	0.0070 (18)	0.0013 (16)	0.0046 (18)
C4	0.026 (2)	0.020 (2)	0.0187 (19)	0.0013 (17)	0.0017 (16)	−0.0013 (15)
C5	0.024 (2)	0.024 (2)	0.019 (2)	0.0049 (16)	−0.0004 (16)	−0.0031 (18)
C6	0.021 (2)	0.023 (2)	0.0128 (18)	0.0052 (17)	0.0003 (15)	0.0013 (15)
C7	0.0197 (19)	0.022 (2)	0.019 (2)	0.0020 (17)	−0.0003 (15)	0.0009 (17)
C8	0.023 (2)	0.029 (2)	0.0188 (18)	0.0036 (17)	0.0015 (16)	0.0010 (17)
C9	0.027 (2)	0.028 (2)	0.023 (2)	0.0004 (18)	0.0009 (17)	0.0042 (18)
C10	0.026 (2)	0.023 (2)	0.031 (3)	0.0001 (17)	0.0013 (19)	−0.0009 (18)
C11	0.033 (2)	0.028 (2)	0.026 (2)	0.001 (2)	0.0016 (19)	−0.004 (2)
C12	0.027 (2)	0.033 (3)	0.0172 (19)	−0.0010 (19)	0.0026 (16)	−0.0004 (18)
C13	0.031 (2)	0.020 (2)	0.031 (2)	0.0041 (19)	−0.0016 (19)	0.0007 (18)
C14	0.029 (2)	0.021 (2)	0.0199 (18)	0.0020 (18)	0.0007 (16)	−0.0028 (17)
C15	0.0170 (17)	0.017 (2)	0.018 (2)	0.0035 (16)	−0.0027 (15)	−0.0001 (16)
C16	0.025 (2)	0.022 (2)	0.0131 (18)	0.0017 (16)	0.0016 (15)	0.0015 (15)
C17	0.0219 (19)	0.025 (2)	0.0174 (19)	0.0030 (16)	0.0019 (15)	−0.0021 (16)
C18	0.0228 (19)	0.019 (2)	0.0196 (19)	0.0006 (16)	−0.0029 (14)	0.0030 (15)
C19	0.026 (2)	0.028 (2)	0.0150 (18)	0.0051 (18)	−0.0014 (16)	−0.0002 (17)
C20	0.028 (2)	0.027 (2)	0.014 (2)	0.0015 (17)	−0.0006 (15)	−0.0017 (15)

### Geometric parameters (Å, º)

**Table:**

Br1—C18	1.944 (4)	C9—H9	0.9500
O1—C2	1.217 (6)	C10—C11	1.354 (7)
N1—C6	1.356 (6)	C10—H10	0.9500
N1—C2	1.456 (6)	C11—C12	1.436 (7)
N1—C7	1.503 (6)	C11—H11	0.9500
N2—C14	1.127 (6)	C12—H12	0.9500
C2—C3	1.485 (7)	C13—H13A	0.9800
C3—C4	1.352 (7)	C13—H13B	0.9800
C3—H3	0.9500	C13—H13C	0.9800
C4—C5	1.464 (7)	C15—C20	1.342 (6)
C4—C13	1.548 (6)	C15—C16	1.389 (6)
C5—C14	1.429 (6)	C16—C17	1.420 (6)
C5—C6	1.433 (7)	C16—H16	0.9500
C6—C15	1.520 (6)	C17—C18	1.329 (6)
C7—C8	1.353 (6)	C17—H17	0.9500
C7—C12	1.360 (6)	C18—C19	1.380 (6)
C8—C9	1.437 (7)	C19—C20	1.417 (6)
C8—H8	0.9500	C19—H19	0.9500
C9—C10	1.371 (7)	C20—H20	0.9500
			
C6—N1—C2	121.0 (4)	C10—C11—H11	119.1
C6—N1—C7	120.1 (4)	C12—C11—H11	119.1
C2—N1—C7	118.6 (3)	C7—C12—C11	121.1 (4)
O1—C2—N1	116.5 (4)	C7—C12—H12	119.5
O1—C2—C3	126.0 (4)	C11—C12—H12	119.5
N1—C2—C3	117.5 (4)	C4—C13—H13A	109.5
C4—C3—C2	123.2 (4)	C4—C13—H13B	109.5
C4—C3—H3	118.4	H13A—C13—H13B	109.5
C2—C3—H3	118.4	C4—C13—H13C	109.5
C3—C4—C5	115.7 (4)	H13A—C13—H13C	109.5
C3—C4—C13	121.7 (4)	H13B—C13—H13C	109.5
C5—C4—C13	122.6 (4)	N2—C14—C5	176.7 (5)
C14—C5—C6	119.2 (4)	C20—C15—C16	116.6 (4)
C14—C5—C4	117.1 (4)	C20—C15—C6	121.9 (4)
C6—C5—C4	123.6 (4)	C16—C15—C6	121.4 (4)
N1—C6—C5	118.9 (4)	C15—C16—C17	123.4 (4)
N1—C6—C15	116.8 (4)	C15—C16—H16	118.3
C5—C6—C15	124.0 (4)	C17—C16—H16	118.3
C8—C7—C12	118.1 (4)	C18—C17—C16	118.8 (4)
C8—C7—N1	118.7 (4)	C18—C17—H17	120.6
C12—C7—N1	123.1 (4)	C16—C17—H17	120.6
C7—C8—C9	120.4 (4)	C17—C18—C19	119.0 (4)
C7—C8—H8	119.8	C17—C18—Br1	118.9 (3)
C9—C8—H8	119.8	C19—C18—Br1	122.1 (3)
C10—C9—C8	122.2 (4)	C18—C19—C20	121.8 (4)
C10—C9—H9	118.9	C18—C19—H19	119.1
C8—C9—H9	118.9	C20—C19—H19	119.1
C11—C10—C9	116.4 (4)	C15—C20—C19	120.4 (4)
C11—C10—H10	121.8	C15—C20—H20	119.8
C9—C10—H10	121.8	C19—C20—H20	119.8
C10—C11—C12	121.8 (5)		
			
C6—N1—C2—O1	176.5 (4)	C2—N1—C7—C12	75.3 (5)
C7—N1—C2—O1	−10.1 (6)	C12—C7—C8—C9	−0.5 (6)
C6—N1—C2—C3	−4.8 (6)	N1—C7—C8—C9	177.2 (4)
C7—N1—C2—C3	168.5 (4)	C7—C8—C9—C10	0.5 (7)
O1—C2—C3—C4	−178.3 (5)	C8—C9—C10—C11	0.2 (7)
N1—C2—C3—C4	3.2 (7)	C9—C10—C11—C12	−0.9 (7)
C2—C3—C4—C5	−0.2 (7)	C8—C7—C12—C11	−0.1 (7)
C2—C3—C4—C13	178.2 (4)	N1—C7—C12—C11	−177.8 (4)
C3—C4—C5—C14	177.5 (4)	C10—C11—C12—C7	0.9 (7)
C13—C4—C5—C14	−0.8 (7)	N1—C6—C15—C20	−117.6 (5)
C3—C4—C5—C6	−1.4 (7)	C5—C6—C15—C20	68.0 (6)
C13—C4—C5—C6	−179.8 (4)	N1—C6—C15—C16	65.0 (6)
C2—N1—C6—C5	3.4 (6)	C5—C6—C15—C16	−109.5 (5)
C7—N1—C6—C5	−169.9 (4)	C20—C15—C16—C17	−0.8 (6)
C2—N1—C6—C15	−171.3 (4)	C6—C15—C16—C17	176.8 (4)
C7—N1—C6—C15	15.4 (6)	C15—C16—C17—C18	1.3 (7)
C14—C5—C6—N1	−179.1 (4)	C16—C17—C18—C19	−0.9 (7)
C4—C5—C6—N1	−0.2 (7)	C16—C17—C18—Br1	179.7 (3)
C14—C5—C6—C15	−4.8 (7)	C17—C18—C19—C20	0.1 (7)
C4—C5—C6—C15	174.1 (4)	Br1—C18—C19—C20	179.5 (3)
C6—N1—C7—C8	71.1 (5)	C16—C15—C20—C19	−0.1 (7)
C2—N1—C7—C8	−102.3 (5)	C6—C15—C20—C19	−177.6 (4)
C6—N1—C7—C12	−111.3 (5)	C18—C19—C20—C15	0.4 (7)

### Hydrogen-bond geometry (Å, º)

**Table:**

*D*—H···*A*	*D*—H	H···*A*	*D*···*A*	*D*—H···*A*
C16—H16···N2^i^	0.95	2.55	3.234 (6)	129
C17—H17···O1^ii^	0.95	2.56	3.342 (6)	140
C20—H20···O1^iii^	0.95	2.40	3.256 (6)	150

Symmetry codes: (i) −*x*+3/2, *y*−1/2, *z*−1/2; (ii) *x*+1/2, −*y*+1/2, *z*; (iii) −*x*+1, −*y*+1, *z*+1/2.
